# Complete mitochondrial genome and phylogenetic position of *Pangasius sanitwongsei* (Siluriformes: Pangasiidae)

**DOI:** 10.1080/23802359.2020.1719915

**Published:** 2020-01-31

**Authors:** Lingjing Wei, Xiangchen Ye, Yejian Lv, Zhongzuo Teng, Baojiang Gan, Hui Zou, Feilong Mo, Sheng Zhang

**Affiliations:** Aquatic Species Introduction and Breeding Center of Guangxi, Nanning, China

**Keywords:** *Pangasius sanitwongsei*, mitochondrial genome, structural organization

## Abstract

In this study, the complete mitochondrial genome of *Pangasius sanitwongsei* was firstly reported and analyzed. It had a double-stand DNA molecule with 16536 bp in length, consisting of 13 protein-coding genes, 2 ribosomal RNA genes, 22 transfer RNA genes and one control region. The structural organization and gene order was similar to other bony fishes. The complete mitochondrial genome of *P. sanitwongsei* provided in this work would be helpful for further research on phylogenetics and conservation genetics of the Siluriformes and other orders.

*Pangasius sanitwongse* (Siluriformes: Pangasiidae) is a large carnivorous freshwater fish, and only be found in southeast Asia, including the lower reaches of the Lancang river,the whole Mekong river and chao phraya river (Dudgeon, 2011; Gray et al. 2017). Now the species is considered as endangered species with limited genetic information reported. Here, the whole mtDNA of *P. sanitwongsei* is cloned and analyzed for the first time, which can be used for species identification and genetic evolution analysis (Mohindra et al. 2015).

Samples was collected from lancang river (100.85 N, 21.98E), Yunnan Province and the specimens were deposited in specimen room of Aquatic species introduction and breeding center of Guangxi (specimen no. YN20190915016). A few tail fin of *P. sanitwongsei* was preserved for total DNA extraction. The protocol of primer design for PCR and data processing method of raw data was according to the method described by Ye et al. (2018). The secondary structure of tRNA genes were analyzed using ARWEN (Laslett and Canback 2008).

The mitochondrial genome of *P. sanitwongsei* was a double-strand circular DNA with the length of 16,536 bp (GenBank accession no. MN809630). Structural organization and gene orders of the mtDNA was consists of 37 parts, including 13 protein-coding genes, 2 ribosomal RNAs, 22 transfer RNAs and one control region, and most of genes located on H strand except for ND6 gene and 8 tRNAs. The overall base composition of the sequence was estimated to be 31.92% A, 15.73% G, 24.86% T, and 27.50% C, with a slight A + T bias (56.78%). Among the 13 protein-coding genes, 12 genes shared start condon ATG except CO1 gene (GTG), 8 genes were terminated with end condons TAA or TAG, while 4 genes (CO2, CO3, ND4 and CYBT) showed the incomplete stop codons (T). 22 tRNA genes could fold into a typical cloverleaf structure, with lengths ranging from 64 bp to 76 bp. The 12S rRNA (948 bp) and 16S rRNA (1677 bp) were flanked by tRNA^Val^, respectively. The control region was flanked by the tRNA^pro^ and tRNA^phe^ genes with 886 bp in length, which was slightly shorter than other closely related species.

Phylogenetic reconstruction based on the complete mitochondrial genome shared by other order species was constructed by the Neighbor-Joining method (NJ) method and NJ bootstrap analysis using MEGA7.0 with 1000 bootstrap replicates (Tamura et al. 2004). Additionally, *Cyprinus carpio* reckoned as the out-group. The phylogenetic result divided the 15 fishes into four groups. Phylogenetic analysis showed that *P. sanitwongsei* clustered to *Pangasius larnaudii* and *Pangasianodon hypophthalmus*, which indicated the phylogenesis classification of *P. sanitwongsei* was consistent with the morphological result (Figure 1).

**Figure 1.**
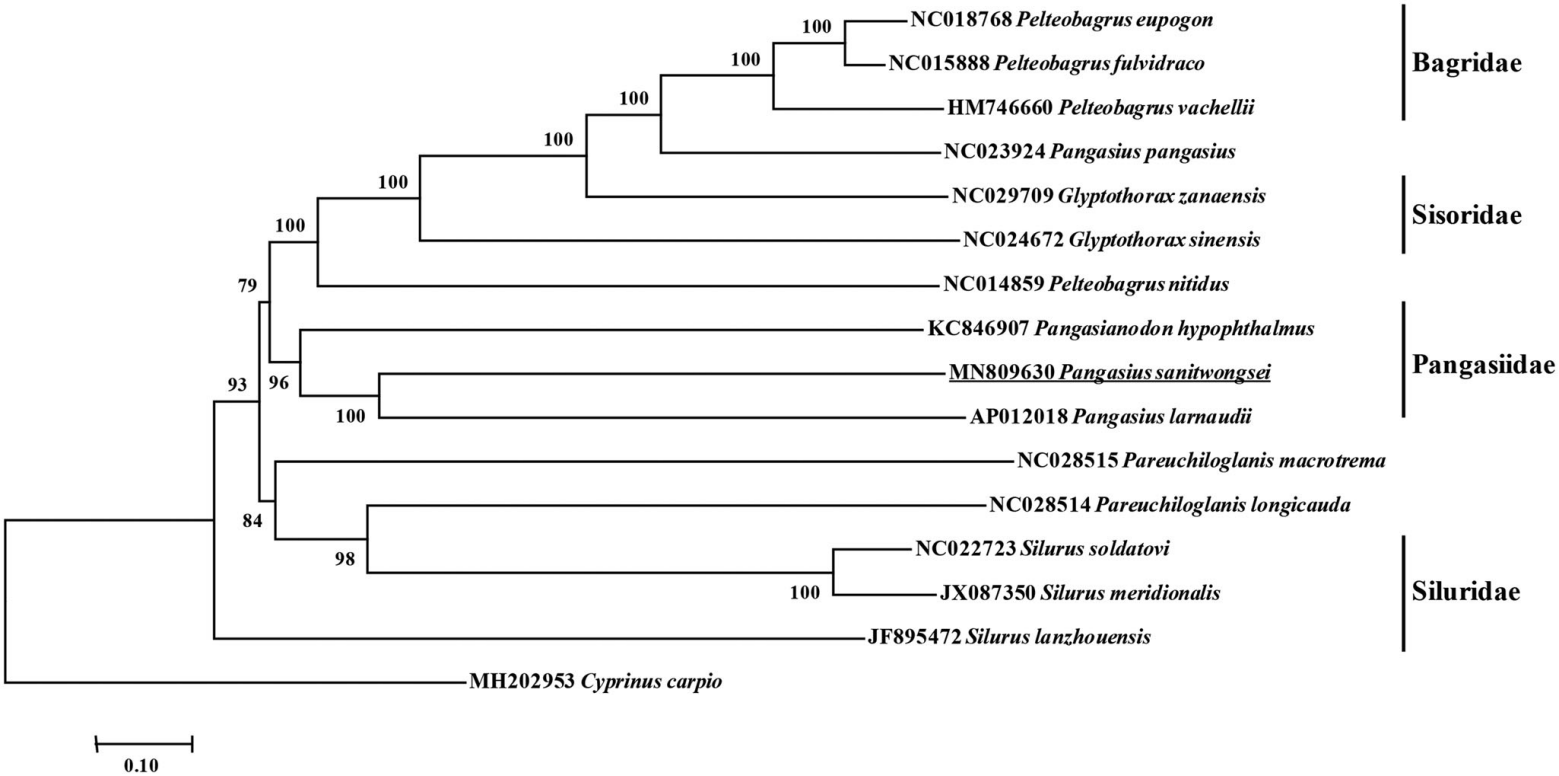
Molecular phylogeny of *Pangasius sanitwongse* and other Siluriformes varieties based on complete mitogenome. The phylogenic tree is constructed by neighbor joining method with 1000 bootstrap replicates. The mtDNA sequences are downloaded from Genbank, The sequence data for phylogenetic analyses used in this study were as follows: *Pelteobagrus eupogon* (NC018768), *Pelteobagrus fulvidraco* (NC015888), *Pelteobagrus vachellii* (HM746660), *Pangasius pangasius* (NC023924), *Glyptothorax sinensis* (NC024672), *Glyptothorax zanaensis* (NC029709), *Pelteobagrus nitidus* (NC014859), *Pangasianodon hypophthalmus* (KC846907), *Pangasius larnaudii* (AP012018), *Pareuchiloglanis macrotrema* (NC028515), *Pareuchiloglanis longicauda* (NC028514), *Silurus soldatovi* (NC022723), *Silurus meridionalis* (JX087350), *Silurus lanzhouensis* (JF895472) and *Cyprinus carpio* (MH202953).

## References

[CIT0001] Dudgeon D. 2011. Asian river fishes in the Anthropocene: threats and conservation challenges in an era of rapid environmental change. J Fish Biol. 79(6):1487–1524. 22136237 10.1111/j.1095-8649.2011.03086.x

[CIT0002] Gray TNE, Phommachak A, Vannachomchan K, Guegan F. 2017. Using local ecological knowledge to monitor threatened Mekong megafauna in Lao PDR. PLoS One. 12(8):e0183247. 28820901 10.1371/journal.pone.0183247 PMC5562319

[CIT0003] Laslett D, Canback B. 2008. ARWEN: a program to detect tRNA genes in metazoan mitochondrial nucleotide sequences. Bioinformatics. 24(2):172–175. 18033792 10.1093/bioinformatics/btm573

[CIT0004] Mohindra V, Singh R K, Kumar R, Sah R S, Lal K K. 2015. Genetic divergence in wild population of endangered yellowtail Catfish Pangasius pangasius (Hamilton-Buchanan, 1822) revealed by mtDNA. Mitochondrial DNA. 26(2):182–186. 24409876 10.3109/19401736.2013.861455

[CIT0005] Tamura K, Nei M, Kumar S. 2004. Prospects for inferring very large phylogenies by using the neighbor-joining method. Proc Natl Acad Sci U S A. 101(30):11030–11035. 15258291 10.1073/pnas.0404206101 PMC491989

[CIT0006] Ye X, Lv Y, Wei L, Huang J, Liu K. 2018. The complete mitochondrial genome of Jinbian carp Cyprinus carpio (Cypriniformes: Cyprinidae). Mitochondrial DNA Part B. 3:1096–1097. 33474430 10.1080/23802359.2018.1495126 PMC7800910

